# The Effect of Unilateral Erector Spinae Plane Block With Dexmedetomidine as an Additive Versus Intrathecal Morphine on Early Mobilization After Total Hip Replacement Under Spinal Anesthesia in Cairo University Hospitals: A Randomized Controlled Trial

**DOI:** 10.1155/anrp/9919921

**Published:** 2026-06-30

**Authors:** Ayman Mohamed Hussam Al-din Abd Al-latif, Ahmed Abdelhamed Hassan, Osama Mohamed Asaad Farahat, Atef Kamel Salama Salem, Karim Hussein Mourad Ghaleb

**Affiliations:** ^1^ Anesthesiology and Intensive Care Medicine Department, Faculty of Medicine, Cairo University, Giza, Egypt, cu.edu.eg

## Abstract

**Background:**

Enhanced recovery after surgery (ERAS) is a current concept in surgery that has excellent short‐term outcomes. Currently, no standardized ERAS protocol exists for total hip replacement (THR). So, we conducted this study to assess the efficacy of ultrasound‐guided erector spinae plane block (ESPB) with dexmedetomidine as an additive in comparison with intrathecal morphine (ITM) for ERAS after THR in Cairo University hospitals.

**Methods:**

Our randomized controlled study included 70 patients. The patients were randomly allocated to the M group or E group. M group patients received 5 mL of subcutaneous lidocaine 1% and then 3.5 mL of hyperbaric bupivacaine 0.5% with 0.1 mg of morphine in 0.25 mL of normal saline intrathecally. E group patients received ESPB with dexmedetomidine as an additive on the side of surgery and then 3.5 mL of hyperbaric bupivacaine 0.5% with 0.25 mL of normal saline intrathecally. The primary outcome was the time of the first successful walking trial for 10 steps with a walker. Secondary outcomes were postoperative pain score, total morphine consumption, duration of hospital stay, patient satisfaction, and incidence of complications (hypotension, bradycardia, pruritus, nausea, vomiting, and urine retention).

**Results:**

The mean time of a successful walking trial with a walker of the E group was 12.69 ± 2.42 h which is decreased significantly in comparison to the M group (19.71 ± 3.11 h), with a mean difference of 7.0 (95% CI 8.81–5.2) hours, with a very large effect size (Cohen^’^s *d* = 2.51), and *p* value < 0.001. The mean number of failed walking trials in the E group (1.11 ± 0.4 trials) decreased to a statistically significant value in comparison to the mean number of failed walking trials in the M group (2.29 ± 0.52 trials), with a mean difference of 1.18 (95% CI 0.96–1.4) trials, and *p* value < 0.001. Duration of hospital stay after surgery was, on average, 33.9% lower in E group in comparison to M group (1.66 ± 0.59 and 2.51 ± 0.51 days, respectively) with a large effect size (Cohen^’^s *d* = 1.54) and *p* value < 0.001. There was no significant difference regarding the mean time of the first morphine dose in M and E groups (16.8 ± 4.78 h and 18.86 ± 4.63 h, respectively) and *p* value = 0.072. Total morphine consumption in the first 24 h was insignificantly higher in the M group than in the E group (7.36 ± 2.97 mg and 5.76 ± 2.88 mg, respectively), with a mean difference of −1.6 (95% CI −2.99 to −0.2) mg and *p* value = 0.025. The patient satisfaction score was better in the E Group than in the M Group. The number of patients with complete satisfaction was more in the E group (24 [68.6%]) than in the M group (10 [28.6%]) and *p* value < 0.001. The rate of complications was higher in the M Group than in E Group (56.19% vs. 22.86%, respectively, with an odds ratio of 4.01 (95% CI, 1.43–11.25) and *p* value = 0.008.

**Conclusion:**

ESPB with dexmedetomidine as an additive enhances early mobilization after THR better than ITM with about a 34% decrease in the duration of hospital stay and a lower rate of complications.

**Trial Registration:** ClinicalTrials.gov Protocol Registration and Results System: NCT06621849

## 1. Introduction

In the new millennium, many countries started enhanced recovery after surgery (ERAS) programs in the early postoperative period after total hip replacement (THR) to allow a shorter hospital stay and rapid ambulation of patients. The outcome of this operation is dependent on the quality of postoperative rehabilitation and the patient’s performance during it [1]. Several patients undergoing joint replacement nowadays are young and need to be rapidly and adequately integrated back into work life.

Although numerous reports have been published on ambulatory and day‐case THR surgery, a standardized multidisciplinary approach has not yet been established. Furthermore, broader evidence‐based acceptance is required to facilitate the widespread implementation of this fast‐track rehabilitation program [2].

Regional anesthesia techniques are a recommended component of multimodal pain management after THR. The hip joint has complex innervation involving branches of the femoral nerve, obturator nerve, and sciatic nerve, while the lateral femoral cutaneous nerve innervates the skin of the lateral side of the thigh. ESPB involves the injection of local anesthetics in the fascial plane between the transverse processes and erector spinae muscles. This new approach has generated tremendous interest and may be applicable to a wide variety of surgeries [3].

Dexmedetomidine as an additive has a well‐documented analgesic and prolonging effect. Also, it has opioid‐sparing properties, which decrease opioid use and prevent opioid side effects [4].

ESPB has been shown to improve pain relief and patient satisfaction after numerous orthopedic surgeries [5]. However, previous studies that discussed this technique after THR had conflicting data. So, further studies are needed.

Intrathecal morphine (ITM) interacts with opioid receptors of the posterior spinal cord, and its hydrophilic nature enables wider spread in CSF with prolonged analgesia [4].

The aim of the study was evaluation of the efficacy of ultrasound‐guided ESPB with dexmedetomidine as an additive in comparison with ITM for ERAS after THR in Cairo University hospitals.

We hypothesized that ESPB has a helpful effect on early mobilization after THR surgery.

## 2. Materials and Methods

### 2.1. Study Design

Our study was an interventional, double‐blind, randomized, and controlled clinical trial.

The study was conducted following approval from the Research Ethics Committee of the Faculty of Medicine, Cairo University (approval number: MD‐259‐2024; date of approval: August 25, 2024), and written informed consent was acquired from all subjects [6]. Patients scheduled prospectively for THR under spinal anesthesia from October 2024 to May 2025.

### 2.2. Eligibility Criteria

All participants were adults 18–60 years old, both genders were allowed, and they had American Society of Anesthesiology classes I or II with BMI between 20 and 30 kg m^−2^ and height between 160 and 190 cm [7]. Exclusion criteria were known allergy to any drug enrolled in the study, any contraindication of spinal anesthesia, urgent surgery, neuromuscular disorder interfering with sensations in the lower limbs, drug abuse or using any drug that modifies pain perception, and any disability affecting walking capacity other than the operating joint.

### 2.3. Study Protocol

Preoperatively, patients who fulfilled inclusion criteria were evaluated by medical history, physical examination, and clinical laboratory tests, which are a complete blood picture (CBC), kidney function tests, liver function tests, international normalized ratio (INR), and chest x‐ray. An electrocardiograph (ECG) was done for patients above 40 years old. Patients were prepared by 8 h of preoperative fasting, receiving a tablet of omeprazole 20 mg and alprazolam 0.5 mg at bedtime the day before surgery. All patients were educated about the standard Numerical Rating Scale (NRS) for pain scoring of 0–10 (0 = *no pain*, 10 = *the most severe pain*) during the pre‐anesthetic evaluation visit.

In our study, 70 patients were randomly divided into two equal groups, with 35 patients in each. *E Group* received ESPB with dexmedetomidine as an additive on the ipsilateral side of the surgery and then 3.5 mL of bupivacaine 0.5% and 0.25 mL of normal saline intrathecally. *M Group* received 5 mL of subcutaneous lidocaine 1%, then 3.5 mL of bupivacaine 0.5% and 0.1 mg of morphine in 0.25 mL of normal saline intrathecally [8].

### 2.4. Study Procedure and Anesthetic Technique

Upon arrival of patients in the operating room, an 18‐gauge intravenous cannula was inserted, and normal saline (10 mL kg^−1^) was infused as a preload. The patients were connected to a monitor to record heart rate (HR), noninvasive measurements of systolic blood pressure (SBP), diastolic blood pressure (DBP), mean blood pressure (MAP), continuous ECG monitoring, and oxygen saturation (SpO_2_). A baseline reading was recorded, and all backup measures and equipment for general anesthesia were ready for urgent use, or rescue general anesthesia could be required.

**FIGURE 1:**
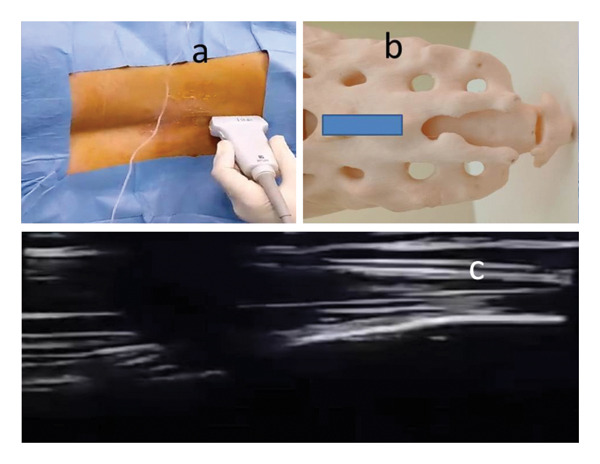
(a) Probe position on the sacral area as a land mark to scan the block site. (b) Simulation of probe position on the sacral area. (c) Ultrasound view of the sacral region with the deepest hyperechoic line representing the sacral periosteum.

**FIGURE 2:**
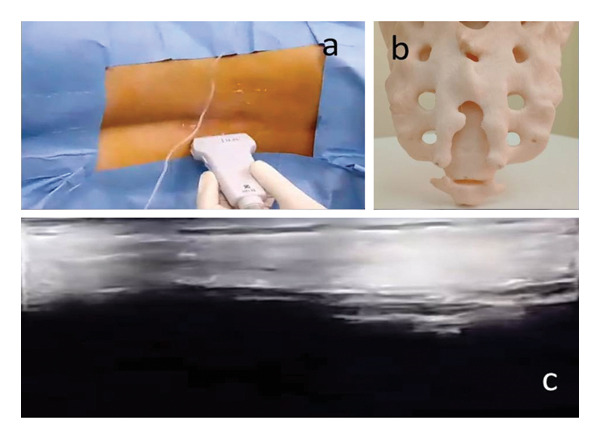
(a) Probe position on the transverse processes of L4 and L5 vertebrae. (b) Simulation of probe position on the transverse processes of L4 and L5 vertebrae. (c) Ultrasound view of the transverse processes of L4 and L5 vertebrae, with the deepest hyperechoic line representing the periosteum of the transverse processes, and above it, the plane of the erector spinae muscle can be seen.

**FIGURE 3:**
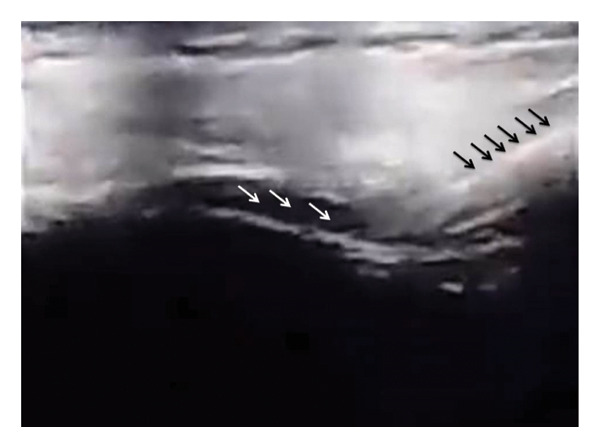
Ultrasound view of the erector spinae plane block in the lumbar region. Black arrows represent the echogenic needle through the in‐plane technique, traversing the muscle layers and reaching the erector spinae plane. White arrows pointed to the local anesthetic (anechoic area) dissecting the plane between the erector spinae muscle and the transverse process.

In the E group, an ultrasound‐guided erector spinae plane block (ESPB) was performed on the ipsilateral side of surgery with the patient in the lateral position. A linear 6–13 MHz ultrasound probe (SONOSITE M‐Turbo, Fujifilm, USA) was positioned vertically, parallel to the spine, and approximately 3–5 cm lateral to the midline [9]. Moving cephalically from the sacrum (Figure 1), we identified the L5, L4, and L3 transverse processes and erector spinae muscles posteriorly (Figure 2). A 21 G and 70 mm long echogenic needle (SonoPlex, Pajunk Medizintechnologie, Germany) was directed in‐plane, and the needle tip was positioned anterior to the erector spinae muscle at the corner of the transverse process. After the initial saline injection, dissection of the plane was observed by injecting a total volume of 20 mL composed of 10 µg of dexmedetomidine in 2 mL of normal saline and 18 mL of bupivacaine 0.25%, which is far from the toxic dose [9]. Correct placement was defined as the spread of local anesthetic cranially and caudally from the injection point (Figure 3), dissecting the plane between the transverse processes and erector spinae muscles [9].

In the M group, patients received a subcutaneous injection of 5 mL lidocaine as a local anesthetic prior to spinal anesthesia. The procedure was performed using the same patient positioning and ultrasound‐guided technique as for the erector spinae plane block to ensure complete blinding of the patients.

Patients in both groups subsequently received spinal anesthesia. Intrathecal administration consisted of 3.5 mL of 0.5% hyperbaric bupivacaine combined with 0.25 mL of normal saline in the E group, or 0.1 mg morphine diluted in 0.25 mL of normal saline in the M group. Intrathecal drugs were administered using a 25‐G Quincke spinal needle (B. Braun Stimuplex Ultra 360, Germany) inserted at the L3–L4 interspace with the patient in the sitting position under complete sterile conditions. Following drug administration, patients in both groups were positioned supine. Sensory block was assessed using a pinprick every 2 min while the patient was in the supine position until the proper level was reached (T10 dermatome), and the Bromage scale was measured to reach Bromage 3 before surgery [10]. Any decrease in HR below 60/min was treated with intravenous atropine (0.01 mg kg^−1^), and any decrease in MAP below 60 mmHg or 20% of the basal reading was treated by fluid bolus and 5 mg intravenous increments of ephedrine.

Failure of ESPB technique: Technique failure was defined as if the correct spread of local anesthetic cranially and caudally from the injection point, dissecting the plane between the transverse processes and erector spinae muscles, was not immediately visualized [9].

Failure of spinal anesthesia: Technique failure was defined as if the T10 sensory level and/or Bromage 3 scale was not reached after 20 min of spinal injection [10].

Those patients with failed study procedures continued their surgery under the established protocol of THR and postoperative analgesia in our hospital (opioid‐based IV PCA).

We assessed 83 patients for eligibility, 13 patients did not fully fit the inclusion criteria, and the remaining 70 patients were randomly allocated in both groups and continued the study till reaching the endpoint.

After finishing the surgery, patients were transferred from the OR to the post‐anesthesia care unit until they reached a modified Aldrete score of ≥ 9, NRS less than 4, and no nausea or vomiting and were able to move the non‐operated limb and then discharged to the ward.

For both groups, oral paracetamol 1 g every 6 h and sustained‐release diclofenac 75 mg every 12 h will be administered postoperatively regularly until hospital discharge. If any patient enrolled in our study complains of a pain score > 3 regarding the numerical rating score (NRS) in spite of the previously mentioned protocol for pain management, a rescue of 0.07 mg kg^−1^ IV morphine sulfate is administered, and then NRS is reassessed 15 min later. If it is still > 3, a rescue 0.05 mg kg^−1^ IV morphine sulfate is administered, which can be repeated any time postoperatively until NRS is 3 or less, provided that total morphine consumption does not exceed 0.2 mg kg^−1^ every 4 h [11]. The previously mentioned IV morphine supplementation protocol was used in pain assessment during rest. If the NRS is more than 3 during the walking trial, we abort the trial and reassess the NRS after 15 min.

### 2.5. End Point

Our study finished with the discharge of the patient from the hospital.

### 2.6. Outcomes

The primary outcome of the study was the time of the first successful trial for walking 10 steps with a walker. In the registry, it was broadly described as “walking without support”; in the manuscript, it is “walking with walker.” This operational definition reflects routine postoperative care for THR and corresponds to the registry’s primary outcome with more safety consideration. All patients were allowed to walk with a walker at 6, 12, 18, and 24 h postoperatively. These trials were conducted after confirming patient safety by following the hospital’s standardized physiotherapy protocol started preoperatively by muscle training, education, and training on walker usage. We followed all the ERAS recommendations from the previous studies regarding the safety of mobilization and walker usage [1–3]. Also, we ensured good and close observation of patients during walking trials by three blinded professional healthcare providers (anesthetist, orthopedic surgeon, and a staff nurse). The trial was aborted if the patient was hemodynamically unstable, still could not move his limb, drowsiness happened during the walking trial, or the NRS was more than 3 during the walking trial.

Secondary outcomes were intraoperative hemodynamics, number of failed walking trials, postoperative pain score, time of first rescue morphine required, total morphine consumption in the postoperative 24 h, duration of hospital stay after surgery, patient satisfaction, and incidence of complications (hypotension, bradycardia, pruritus, nausea, vomiting, and urine retention). So, before every walking trial, a blinded staff nurse checked ABP and pulse and asked the patient about pruritus, nausea, vomiting, and inability to urinate in spite of desire.

### 2.7. Protocol Amendments After Registration

The primary outcome in the registry was walking without support. Before starting the study work, after the trial registration and before randomization of patients, the orthopedic surgery head of department contacted our research supervisor and discussed this modification with us, and the decision was to make it with a walker to put patient safety above all else. Regarding height range, we wrote 160–180 cm in the registry. We found that a lot of our cases’ heights measured between 180 and 190 cm. So, we reviewed the literature regarding the effect of patient height on the level of spinal anesthesia and analgesia to know if we have to adjust the volume of bupivacaine in taller patients, and we found that there is no need for adjusting the dose [7]. So, we accept those taller cases.

### 2.8. Sample Size

As no previous clinical study mentioned our primary outcome or included it in its statistical analysis, we performed a pilot study with 15 patients in each group with the same primary endpoint (the time of the first successful trial for walking 10 steps with a walker). After confirmation of the normality assumption by the Shapiro–Wilk test, we used an independent sample *t*‐test to calculate the mean ± standard deviation for the E group and the M group. The results were (14.61 ± 5.13) and (18.24 ± 3.41), respectively [12]. Epi‐calc 2000 was used to calculate the sample size of this randomized clinical trial. Assuming 90% study power with a strong effect size (Cohen’s *d* = 0.83), a type I error of 0.05, a mean difference of 3.6 (95% CI 6.88–0.37) hours, and a pooled standard deviation calculated of ± 4.36 h. The calculated number was 31 patients for each group; as we had a two‐limb study, the total sample was 62 subjects. The number of envelopes was increased by 10% to compensate for possible dropouts, so the sample size was 70 participants (35 in each group). Data from the pilot study were included in the final analysis.

### 2.9. Randomization

A computer‐generated sequence was used for randomization before induction of anesthesia. Patients were allocated sequentially to their groups as per numbered opaque envelopes.

### 2.10. Blinding

The ethical committee of our institute refused a sham block of ESPB, so we established this blinding method. Firstly, allocation concealment was done by central randomization. The patients were blinded as they did not know the additive for intrathecal bupivacaine and the type of block (either ESPB or subcutaneous lidocaine). A researcher who was blinded to group allocation and study design prepared sealed opaque envelopes. On the day of surgery a consultant anesthesiologist, who was not involved in data collection or analysis, opened the envelopes and started ESPB or subcutaneous lidocaine injection. This consultant prepared additives of spinal anesthesia, the ITM or normal saline, in a syringe, and another anesthesiologist, who was blinded to the block and additive, performed the spinal technique. Intraoperative data were collected by the anesthesiologist who performed spinal anesthesia. Surgeons did not attend any part of the anesthetic technique. Recovery room researchers and inpatient researchers that collected the postoperative data were not aware of the allocated patients’ group. Finally, the statistician received the data as a Group A sheet and a Group B sheet without reference to ESPB or ITM. So, the patient, the anesthetist who performed the SA, the surgeons, the data collectors, and the statistician were blinded. This strict blinding protocol was unmasked only after completion of data analysis. Only the consultant anesthesiologist, who opened the envelopes, was aware of the block and additive of SA; therefore, he was not allowed to attend any intraoperative or postoperative data collection or analysis and had no contact with the patients.

We tried hard to confirm complete blinding, but we admitted that blinding may not have been fully secure at the patient level.

### 2.11. Statistical Method

Data were collected, coded, tabulated, and statistically analyzed using the SPSS program (Statistical Package for Social Sciences) software Version 24.0. Data were summarized using mean and standard deviation for normally distributed quantitative variables or median and interquartile range for non‐normally distributed quantitative variables and number of cases and percentages for categorical variables. The Shapiro–Wilk test was used to test the normality of all continuous variables. Comparisons between groups were done using an independent sample *t*‐test for normally distributed quantitative variables, while the Mann–Whitney test was used for non‐normally distributed quantitative variables. For comparing categorical data, the chi‐square test was used. *p* values less than 0.05 were considered statistically significant. The confidence interval was 95% for all main outcomes.

## 3. Results

**FIGURE 4:**
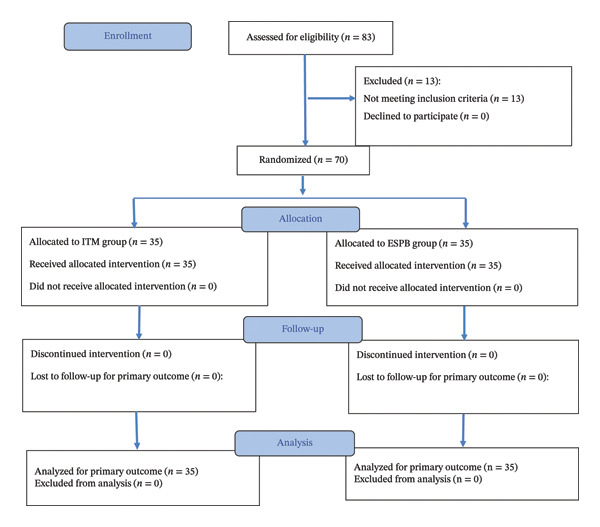
Consolidated standards of reporting trials (CONSORT) flow diagram. Abbreviations: n, number; ITM, intrathecal morphine; ESPB, erector spinae plane block.

**FIGURE 5:**
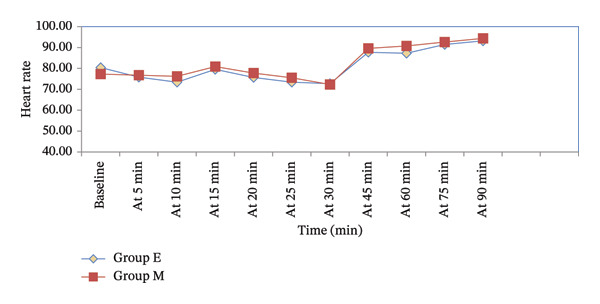
Comparison between the two groups according to heart rate using the line graph. Abbreviations: min, minutes.

**FIGURE 6:**
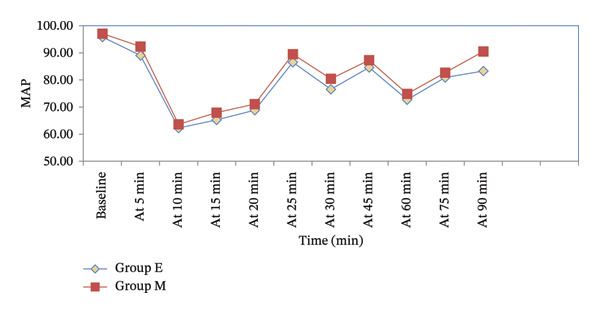
Comparison between the two groups according to MAP using the line graph. Abbreviations: MAP, mean arterial pressure, min, minutes.

**FIGURE 7:**
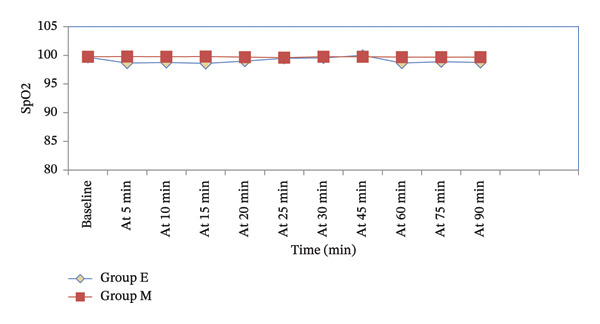
Comparison between the two groups according to SpO_2_ using the line graph. Abbreviations: SpO_2_, oxygen saturation; min, minutes.

**FIGURE 8:**
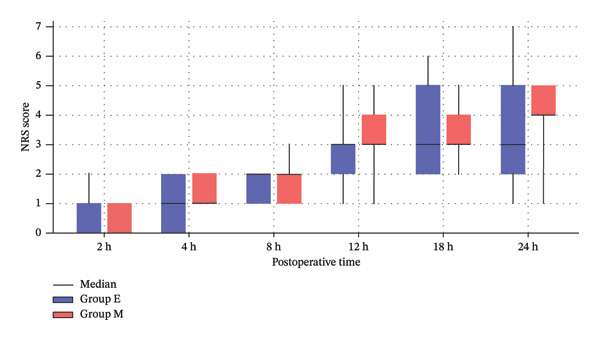
Comparison between the two groups according to postoperative NRS using a paired box‐and‐whisker plot graph. Abbreviations NRS, Numerical Rating Scale; h, hours.

Our study adheres to the applicable CONSORT guidelines from enrollment to analysis (Figure 4). During the study period, 70 patients enrolled in our study out of a total of 83 patients assessed for eligibility. The 70 patients (35 in each group) continued the study until reaching the end point. Demographic data and ASA classification were comparable between groups (Table 1). Regarding the primary outcome (time of successful walking trial with a walker) of the E group, it was 12.69 ± 2.42 h, which is decreased significantly in comparison to the M group (19.71 ± 3.11 h), with a mean difference of 7.0 (95% CI 8.81–5.2) hours, a *p* value < 0.001 (Table 2) with a very large effect size (Cohen’s *d* = 2.51). Although this effect size is unusually large for a small single‐center trial, it may indicate a clinical plausibility and refer to the exploratory nature of our findings. Regarding secondary outcomes, we did 5 independent sample *t*‐tests with type I errors (α value = 0.05). An independent sample *t*‐test was done for intraoperative hemodynamics, number of failed walking trials, time of first rescue morphine required, total morphine consumption in the postoperative 24 h, and duration of hospital stay after surgery. We used the Bonferroni correction calculator to make corrections for multiple comparisons, and alpha became 0.01. Intraoperative hemodynamics were comparable in both groups, including HR (Figure 5), MAP (Figure 6), and SpO_2_ (Figure 7). We found that the mean number of failed walking trials in the E group (1.11 ± 0.4 trials) decreased to a statistically significant value in comparison to the M group (2.29 ± 0.52 trials), a mean difference of 1.18 (95% CI 0.96–1.4) trials, with a very large effect size (Cohen’s *d* = 2.55) and a *p* value < 0.001 (Table 2). NRS, in the first postoperative 24 h, of both groups was comparable except in the following two times (it was significantly higher in the M group in comparison to the E group): 4 h (1 [1.00–2.00] and 1 [0.00–2.00], respectively, *p* value = 0.006) and 12 h (3 [3.00–4.00] and 3 [2.00–3.00], respectively, *p* value = 0.004) (Figure 8). Duration of hospital stay after surgery was, on average, 33.9% lower in E group in comparison to M group (1.66 ± 0.59 and 2.51 ± 0.51 days, respectively) with a large effect size (Cohen’s *d* = 1.54) and a *p* value < 0.001 (Table 3). There was no significant difference regarding the mean time of the first morphine dose in M and E groups (16.8 ± 4.78 h and 18.86 ± 4.63 h, respectively, *p* value = 0.072). The total morphine consumption in the first 24 h postoperatively was insignificantly higher in the M group than in the E group (7.36 ± 2.97 mg and 5.76 ± 2.88 mg, respectively), with a mean difference of −1.6 (95% CI −2.99 to −0.2) mg, with a moderate effect size (Cohen’s *d* = 0.55) and *p* value = 0.025 (Table 4). Patient satisfaction score was better in the E group than in the M group (Table 3). The rate of complications in the first postoperative 24 h was higher in the M Group than in the E Group (56.19% vs. 22.86%, respectively, with an odds ratio of 4.01 (95% CI, 1.43–11.25) *p* value = 0.008 (Table 3).

**TABLE 1:** Comparison between the two groups according to demographic data and ASA classification.

Demographic data	Group E (*n* = 35)	Group M (*n* = 35)	*p* value^a^ ^,^ ^b^
Age (years) mean (standard deviation)	46.43 (10.87)	47.31 (10.75)	0.865^a^
Sex number (percentage)			
Male	24 (68.6)	21 (60)	0.454^b^
Female	11 (31.4)	14 (40)	
Height (cm) mean (standard deviation)	178.1 (3.8)	177 (5.1)	0.215^a^
BMI (kg m^−2^) mean (standard deviation)	25.85 (3.13)	25.7 (3.17)	0.839^a^
ASA classification number (percentage)			
I	14 (40)	19 (54.3)	0.231^b^
II	21 (60)	16 (45.7)	

*Note: n*, number.

Abbreviations: ASA, American Society of Anesthesiology; BMI, body mass index.

^a^ *p* value of *t*‐test used to compare means.

^b^ *p* value of the chi‐square test used to compare proportions.

**TABLE 2:** Comparison between the two groups according to time of 1^st^ successful walking trial (h) and number of failed trials.

Time of 1^st^ successful walking trial (h) and number of failed trials	Group E (*n* = 35): mean ± standard deviation	Group M (*n* = 35): mean ± standard deviation	*p* value^a^
Time of 1^st^ successful walking trial (h)	12.69 ± 2.42	19.71 ± 3.11	0.001^a^
Number of failed walking trials	1.11 ± 0.4	2.29 ± 0.52	0.001^a^

*Note: n*, number; 1^st^, first.

^a^ *p* value of *t*‐test used to compare means.

**TABLE 3:** Comparison between the two groups according to adverse effects, duration of hospital stay, and patient satisfaction.

	Group E (*n* = 35)	Group M (*n* = 35)	*p* value^a^ ^,^ ^b^
Adverse effects number (percentage)			
Hypotension	8 (22.9)	9 (25.7)	0.781^b^
Bradycardia	2 (5.7)	3 (8.6)	0.643^b^
Pruritus	1 (2.8)	10 (28.57)	0.003^b^
Nausea	7 (20)	16 (45.7)	0.022^b^
Vomiting	4 (11.4)	13 (37.1)	0.012^b^
Urine retention	2 (5.7)	8 (22.9)	0.041^b^
Hospital stay (days) mean ± standard deviation	1.66 ± 0.59	2.51 ± 0.51	0.001^a^
Patient satisfaction number (percentage)			
Complete satisfaction	24 (68.6)	10 (28.6)	0.001^b^
Partial satisfaction	10 (28.6)	23 (65.7)	
No satisfaction	1 (2.8)	2 (5.7)	

*Note: n*, number.

^a^ *p* value of *t*‐test used to compare means.

^b^ *p* value of the chi‐square test used to compare proportions.

**TABLE 4:** Comparison between the two groups according to the time of the 1^st^ rescue morphine (h) and total morphine consumption (mg).

Time of 1^st^ rescue morphine (h) and total morphine consumption (mg)	Group E (*n* = 35): mean ± standard deviation	Group M (*n* = 35): mean ± standard deviation	*p* value^a^
Time of 1^st^ rescue morphine (h)	18.86 ± 4.63	16.8 ± 4.78	0.072^a^
Total morphine consumption (mg)	5.76 ± 2.88	7.36 ± 2.97	0.025^a^

*Note: n*, number; 1^st^, first; mg, milligram.

^a^ *p* value of *t*‐test used to compare means.

## 4. Discussion

ESPB in THR has been demonstrated as a good option for postoperative analgesia through its mechanism of action, including cranio‐caudal spread to all lumbar plexuses and potential diffusion to paravertebral and epidural spaces [13]. It also has a motor‐sparing effect on quadriceps femoris if the used local anesthetic is at its analgesic concentration, which enhances ERAS and enables early discharge and surgery‐day mobilization [14]. When this block is performed with only bupivacaine, its effect can last only for 6–8 h. Dexmedetomidine is a new additive for a lot of regional blocks that prolong the duration of local anesthetics and has analgesic properties [5]. So, we added dexmedetomidine to the bupivacaine to prolong its duration of action and enhance the net analgesic effect.

Regarding our primary outcome, we review the literature to find a previously validated outcome that can explain the effect of the different modalities of pain control on ERAS. We did not find a standard outcome for ERAS. So, we chose our outcome as a pragmatic institute‐specific functional surrogate that depends not only on pain control but also on sedation, motivation, staffing, timing of attempts, and local physiotherapy workflow.

### 4.1. Hemodynamics

Regarding hemodynamic measurements all over the study time, there was no statistically significant difference between the study groups. Our result is going in line with Hamed et al. [15], who conducted their study on 140 female patients scheduled for elective LSCS to compare the analgesic effect of ITM versus ESPB.

On the contrary to our study result regarding hemodynamics, the study carried out by Kaya et al. [16] studied the effects of the ITM and ESPB on postoperative analgesia in 40 patients undergoing video‐assisted thoracoscopic surgeries. They found that main arterial blood pressure was significantly lower in the ITM group in comparison to the ESPB group. But the mentioned study authors used a very high dose of morphine (5 μg kg^−1^) in comparison to our study. This may indicate that higher ITM doses are associated with an increased incidence of hypotension and bradycardia.

Another study conducted by Lal et al. [17] compared ITM versus ESPB for perioperative analgesia in 74 patients undergoing lumbar spine surgery. They found that HR was significantly higher in the ESPB group in comparison to the ITM group. But when we searched on their research methodology, we found that the dose of the ITM was 300 μg, which is more than our study dose. This high dose of ITM was responsible for a higher Ramsay sedation scale score in comparison to the patients in the ESPB group, which was statistically significant and delayed the mobilization of the patients. Not only was the Ramsay sedation scale score, but also the incidence of complications such as oxygen desaturation and constipation was significantly higher in the ITM group in comparison to the ESPB group.

### 4.2. Time of First Successful Walking Trial and Number of Failed Walking Trials

Although there were no previous studies in the literature that compared this outcome, frankly, we found that Meylan et al. [18] mentioned the effect of ITM on movement NRS in his study. This study discussed the benefit and risk of ITM without local anesthetic in patients undergoing major surgery. They concluded that ITM decreases movement NRS significantly. Another study conducted by Zhao et al. [19] mentioned the effect of ITM and ESPB on movement NRS. This study discussed the efficacy of lumbar ESPB for postoperative analgesia management in patients undergoing lumbar unilateral bi‐portal endoscopic surgery. They concluded that ESPB decreases movement NRS significantly.

But unfortunately, during the review of the literature, we did not find a previous study that compared ITM–ESPB in THR regarding movement NRS.

### 4.3. Postoperative Pain Score

During our research study, the decrease of median NRS of group E was statistically significant in comparison to group M in the following two times, 4 h postoperatively and 12 h postoperatively. On the other hand, there was no statistically significant difference between the two groups regarding the median NRS measured at other times (2, 8, 18, and 24 h postoperatively).

Our study results went in line with Hamed et al. [15], who found that there was no statistically significant difference between the ESPB group and the ITM group regarding pain score. Although the VAS was insignificantly higher in the ITM group.

In contrast to our study, Kaya et al. [16, 17] found that the pain score was significantly higher in the ESPB group in comparison to the ITM group. This difference may be due to the high dose of ITM used in those studies in comparison to our study and Hamed et al.’s study.

It is deserved to be mentioned that ITM in Kaya et al. [16] and Lal et al. [17] studies was injected with normal saline and without any local anesthetic. On the other hand, ITM in Hamed et al.’s study, as well as our study, was injected with local anesthetic, 0.5% hyperbaric bupivacaine.

### 4.4. Time of the First Rescue Morphine and Total Morphine Consumption

When we compared the mean time of the first morphine dose in the E and M groups, we found that there was no statistically significant difference between both groups. Regarding the total morphine consumption during the postoperative 24 h, there was no statistically significant difference between the E and M groups.

A study conducted by Fredrickson and Danesh‐Clough [20] compared spinal anesthesia with adjunctive ITM versus continuous lumbar plexus blockade for analgesia after hip replacement on 50 patients. They concluded that patients in the spinal group with ITM required rescue IV morphine more than patients in the other group of the study.

### 4.5. Duration of Hospital Stay After Surgery

As regards our study results, patients were allowed to be discharged from the hospital after they met the discharge criteria. We found that patients of the E group stayed in the hospital a shorter time than patients of the M group by a statistically significant period.

As per the review done by Gonvers et al. [21], who wrote a meta‐analysis on the efficacy and safety of ITM for analgesia after lower arthroplasty. They found that ITM shortened the hospital stay in comparison to control groups, which were not applied to any added regional technique rather than ordinary spinal anesthesia.

Unfortunately, there was no clear data in the literature review on the difference between ESPB and ITM used for THR regarding the duration of hospital stays.

### 4.6. Overall Patient Satisfaction

In the current study, we compared both groups regarding patient satisfaction. There was a statistically significant difference between patients of the E group and patients of the M group, with superiority of ESPB over ITM.

Our study result went in line with Hamed et al. [15], who found that patients of the ESPB group who had excellent satisfaction were more than those in the ITM group with a statistically insignificant difference.

Regarding the results of Kaya et al. [16], they found that patients of the ITM group who were very satisfied were more than those in the ESPB group, but the difference between the two groups was statistically insignificant.

As shown above, there is controversial data regarding patient satisfaction. This can be attributed to the fact that participant satisfaction is a complex phenomenon and does not depend on pain control alone.

### 4.7. Incidence of Complications and Adverse Effects

In our study we found that there was no statistically significant difference between the E group and the M group regarding bradycardia and hypotension. On the other hand, there was a statistically significant decrease in the E group in comparison to the M group regarding the incidence of pruritus, nausea, vomiting, and urine retention.

Our study results were went in line with Lal et al. [17]; in this study, the ITM group had more incidences of complications than the ESPB group, and it was statistically significant.

Fredrickson and Danesh‐Clough [20] reported that the spinal group patients with ITM reported pruritus more than the lumbar plexus block group patients. Antiemetic requirements, episodes of disorientation, and arterial oxygen desaturation were comparable between the two groups.

Another study was conducted by Sibanyoni et al. [22], who studied the use of ITM for acute postoperative pain in lower limb arthroplastic surgery. They compared 100 μg ITM versus 150 μg ITM. They concluded that, although the side effect profile difference was statistically not significant, the percentage of patients who had both pruritus, nausea, and vomiting as side effects was higher in the 150 μg ITM group compared to the 100 μg ITM group. These results showed clearly the rising side effect percentage with the rising ITM dose.

Hamed et al. [15], observed no side effects in their study. No nausea or vomiting was noted. They also added that this result may be due to the premedication with 1 mg of granisetron administered to all participants. No urine retention was recorded, as all participants were catheterized using a Foley catheter. They also did not observe any pruritus or respiratory depression.

Finally, the incidence of side effects and complications had a lot of factors that change the percentage and the significance of occurrence in the different studies. Those factors included, for example, the dose of ITM, type of surgery, the compared group versus ITM, and the premedication protocols before anesthetic procedures.

### 4.8. Limitations

Limitations of the study were a single‐center design, relatively small sample size, lack of long‐term outcomes, limited age range and study population, use of pilot data in final analysis, and possible limitations of the blinding protocol. Postregistration protocol modifications were also limitations. The addition of dexmedetomidine as an additive may be considered a confounder.

## 5. Conclusion

Based on our findings, we concluded that ESPB with dexmedetomidine as an additive enhances early patient mobilization after THR surgery better than ITM. There was a significant decrease in the duration of hospital stays after surgery. There were comparable hemodynamics and pain control in most study times till the end point. There was better patients’ overall satisfaction in the ESPB group with a significantly higher incidence of side effects in the ITM group.

## Funding

No funding was received for conducting this study or supporting its findings.

## Conflicts of Interest

The authors declare no conflicts of interest.

## Data Availability

The data used in this study are available on reasonable request from the authors.
